# A Special Tear Pattern of Anterior Horn of the Lateral Meniscus: Macerated Tear

**DOI:** 10.1371/journal.pone.0170710

**Published:** 2017-01-26

**Authors:** Jiapeng Zheng, Wenliang Zhai, Qiang Li, Qianxin Jia, Dasheng Lin

**Affiliations:** 1 Department of Orthopaedic Surgery, the Affiliated Southeast Hospital of Xiamen University, Zhangzhou, China; 2 Department of Radiology, the Affiliated Southeast Hospital of Xiamen University, Zhangzhou, China; 3 Experimental Surgery and Regenerative Medicine, Department of Surgery, Ludwig-Maximilians-University (LMU), Munich, Germany; Harvard Medical School/BIDMC, UNITED STATES

## Abstract

**Background:**

We describe a special, interesting phenomenon found in the anterior horn of the lateral meniscus (AHLM): most tear patterns in the AHLM are distinctive, with loose fibers in injured region and circumferential fiber bundles were separated. We name it as macerated tear. The goal of this study was to bring forward a new type of meniscal tear in the AHLM and investigate its clinical value.

**Materials and Methods:**

AHLM tears underwent arthroscopic surgery from January 2012 to December 2014 were included. Data regarding the integrity of AHLM were prospectively recorded in a data registry. Tear morphology and treatment received were subsequently extracted by 2 independent reviewers from operative notes and arthroscopic surgical photos.

**Results:**

A total of 60 AHLM tears in 60 patients (mean age 27.1 years) were grouped into horizontal tears (n = 15, 25%), vertical tears (n = 14, 23%), complex tears (n = 6, 10%), and macerated tears (n = 25, 42%). There were 6 patients with AHLM cysts in macerated tear group and one patient in vertical tear group. 60 patients were performed arthroscopic meniscus repairs and were followed-up with averaged 18.7 months. Each group had significant postoperative improvement in Lysholm and IKDC scores (p < 0.05). However, the macerated tear group showed least functional recovery of Lysholm and IKDC scores compared to other groups (p < 0.05). In addition, there were no differences in postoperative range of motion, return to work, or return to sport/other baseline activities between the four groups (p > 0.05).

**Conclusions:**

This study demonstrated that the macerated tear is common in the tear pattern of AHLM. However, feasibility of the treatment of this type of meniscal tear, especially the meniscus repairs still requires further study.

## Introduction

Meniscal tears are very common and are one of the most frequent indications for knee arthroscopy [1–3]. When a meniscal tear is identified, accurate description and classification of the tear pattern can guide the referring clinician in patient education and surgical planning. It is common to classify the injury types according to the direction and morphology of meniscal tears. In most cases, the tear types are divided into vertical, horizontal and complex tears [4–6]. The vertical tears consist of longitudinal, radial and bucket handle tear, while the horizontal tears include transverse or cleavage tear, and the tears combining two or more types are called as complex tears. This classification method is adopted by the surgeons all over the world and taken as the communication basis. However, we found a special, interesting phenomenon in the anterior horn of the lateral meniscus (AHLM): most tear patterns in the AHLM are distinctive, with loose fibers in injured region and circumferential fiber bundles were separated. We name it as macerated tear and define it to be three and more circumferential fiber bundles can be separated by arthroscopic probe at meniscus surface or inside the injury. The goal of this study was to describe a new type of meniscal tear in AHLM from a consecutive series of arthroscopic surgeries.

## Materials and Methods

### Participants

This study was carried out in accordance with the guidelines of the Declaration of Helsinki. All experimental protocols were approved by our institutional review board (Xiamen University Ethical Review Committee, Ethics number: EC-11-012), and written informed consent was obtained from all study participants. From January 2012 to December 2014, we enrolled the patients underwent arthroscopic surgery due to the AHLM tears. Exclusion criteria were the knee ligament injuries (anterior cruciate ligament, posterior cruciate ligament, medial collateral ligament and lateral collateral ligament, et cetera), cartilage lesion visualized on magnetic resonance imaging (MRI), severe synovitis and the patients with medical history of ipsilateral knee joint surgery.

### Treatment

All patients underwent 3 T MRI examinations by a single medical imaging technician and arthroscopic surgery by a single orthopedic surgeon [7]. The knee joint symptoms, signs, Lysholm scores and International Knee Documentation Committee (IKDC) scores were recorded. After MRI and arthroscopic examination, data regarding the integrity of AHLM were prospectively recorded in a data registry. Tear morphology and treatment received were subsequently extracted by 2 independent reviewers from operative notes and arthroscopic surgical photos.

### Statistical analysis

SPSS 19.0 (SPSS company, America) statistical software package was taken for statistical analysis. p < 0.05 indicates significant difference, while p < 0.01 refers to extremely significant difference.

## Results

From January 2012 to December 2014, a total of 1016 knee arthroscopies were performed. A total of 64 AHLM tears were identified in 64 patients (55 male, 9 female) with a mean age of 26.9 years (range, 18–45 years). Of the 64 AHLM tears, 4 AHLM tears were excluded. Despite several contacts by mail and telephone, 3 patients did not attend the clinical visit. One patient with tibial shaft fracture underwent intramedullary nail fixation in the 7th postoperative month. After these exclusions, a total of 60 AHLM tears in 60 patients remained to be study.

In the 60 patients of AHLM tears, there were 52 male patients and 8 female patients, aging from 18 to 45 years with a mean age of 27.1 years, including 20 patients of left knees and 40 patients of right knees. All patients had anterolateral knee pain and hyperextension pain but no knee locking or snap, and physical examination indicated tenderness in lateral gap of anterolateral knee with positive McMurray.

The AHLM tears were organized into 4 distinct types based on tear morphology. Type I tears were defined as horizontal tears with tears in the horizontal axis of the meniscus. Type II tears were defined as vertical tears with tears along the longitudinal axis of the meniscus. Type III tears were defined as complex tears with tears combining horizontal and vertical tears. Type IV tears were defined as macerated tears with loose fibers in injured region and circumferential fiber bundles were separated. Using this classification, 15 patients (25%) were Type I, 14 (23%) were type II, 6 (10%) were type III, and 25 (42%) were type IV. There were 6 patients with AHLM cysts in the macerated tear group and one patient in the vertical tear group. No significant differences in baseline characteristics were found between the four groups. All patients were observed clinically for an averaged 18.7 months (range, 12–36 months) (Table 1).

**Table 1:** Patient demographic information for each group included in the AHLM.

Parameter	Macerated tears	Horizontal tears	Vertical tears	Complex tears
**Overall, n (%)**	25 (42)	15 (25)	14 (23)	6 (10)
**Male/ female, n**	22/3	13/2	12/2	5/1
**Mean age (range), yr**	25.8±5.2 (18–45)	27.8±6.1 (21–43)	28.4±4.9 (19–45)	28.7±4.5 (26–41)
**Right/ left knee involved, n**	16/9	10/5	10/4	4/2
**BMI at surgery, kg/cm2**	24.4±2.7 (21.5–26.3)	23.9±2.4 (21.2–25.8)	24.2±2.9 (21.5–26.8)	24.7±3.1 (22.2–27.6)
**Cyst, n**	6	0	1	0
**Mean follow-up (range), mo**	19.2±3.7 (15–36)	17.9±3.3 (12–36)	18.9±2.6 (15–36)	18.2±2.1 (12–36)
**Lysholm score**				
** Preoperative (range)**	56.5±12.3 (41–68)	57.1±11.7 (43–68)	56.8±12.6 (41–68)	56.4±11.7 (44–64)
** Final follow-up (range)**	83.9±14.8 (76–90)*	89.2±15.3 (80–93)	89.6±14.9 (80–93)	88.4±13.3 (82–93)
**IKDC score**				
** Preoperative (range)**	52.7±13.4 (36–66)	53.2±13.6 (40–66)	52.6±13.1 (40–65)	52.2±12.4 (43–66)
** Final follow-up (range)**	79.8±12.5 (72–90)*	87.4±12.8 (78–93)	87.8±12.2 (80–93)	87.5±11.9 (82–91)
**Postoperative ROM**				
** Mean knee extension (°)**	0	0	0	0
** Mean knee flexion (range), (°)**	121 (95–130)	126 (100–135)	125 (100–135)	123 (95–130)
**Postoperative return to work, n (%)**	25 (100)	15 (100)	14 (100)	6 (100)
**Postoperative return to sport, n (%)**	21/25 (84)	13/15 (87)	12/14 (86)	5/6 (83)

* p < 0.05.

BMI, body mass index; IKDC, International Knee Documentation Committee; ROM, range of motion.

**Fig 1:**
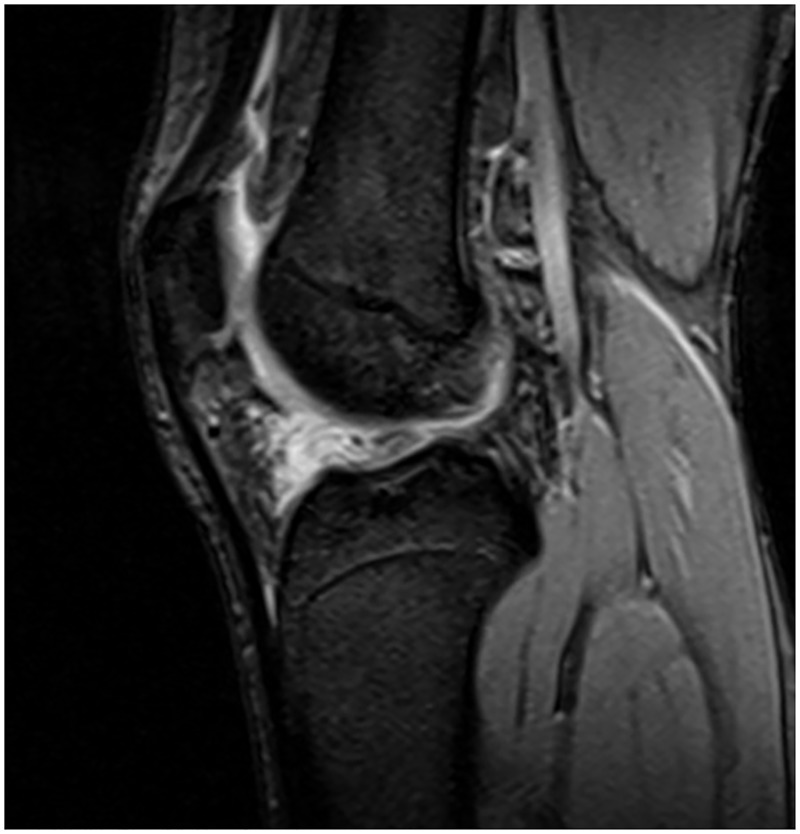
Sagittal proton density-weighted MRI of the knee of a 21-year-old male show that the disorder and irregular strip-like signal was observed in the AHLM and articular capsule.

**Fig 2:**
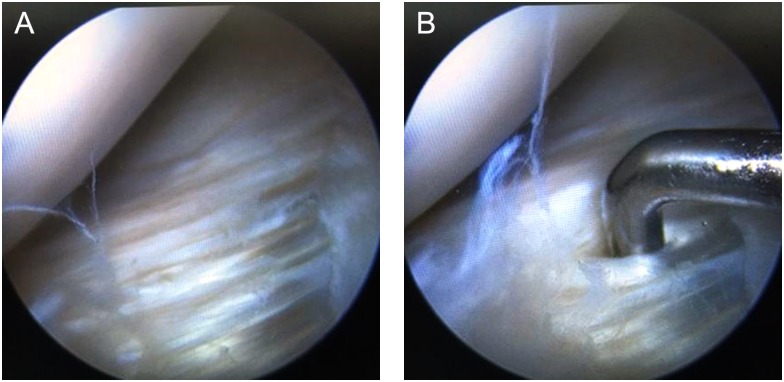
Arthroscopic images show that the AHLM are tears, with loose fibers in injured region and circumferential fiber bundles were separated (A), which can be separated by arthroscopic probe at meniscus surface or inside the injury (B).

In the macerated tear group, there were 22 male patients and 3 female patients, aging from 18 to 45 years with a mean age of 25.8 ± 5.2 years, including 9 cases of left knees and 16 cases of right knees. For the macerated tears, MRI sagittal position T2 sequence showed that disorder and irregular strip-like signal was observed in AHLM and articular capsule, which is connected with articular cavity, but the overall morphology of meniscus is complete without any inversion or dislocation (Fig 1). In the macerated tears with cysts, many circular-like high signals at different sizes around the body and cysts are visible, but the overall morphology of meniscus is complete. Furthermore, for the macerated tears, arthroscopy showed that loose fibers in injured region and circumferential fiber bundles were separated, three and more circumferential fiber bundles can be separated by arthroscopic probe at meniscus surface or inside in AHLM (Fig 2, S1 Video). For the macerated tears with cysts, arthroscopy showed that the cysts are distributed in the circumferential fiber bundles.

**Fig 3:**
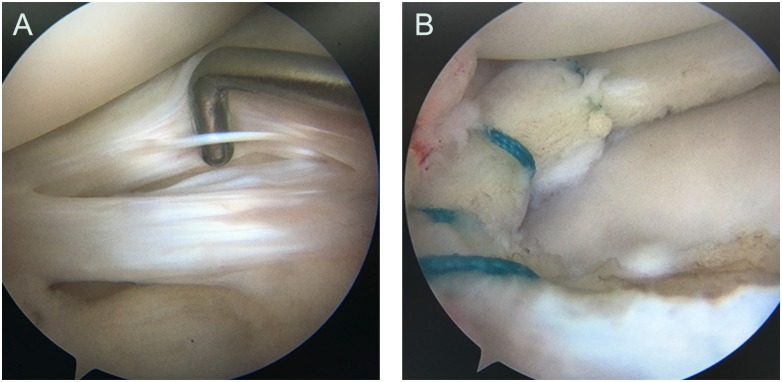
A 18-year-old male with macerated tear of the AHLM (A), and the suture repairs, inside-out horizontal mattress meniscal repairs, were performed treatment (B).

In the four groups, meniscal tears were treated with arthroscopic meniscus suture repairs, and meniscal tears with cysts were performed with arthroscopic excision and suture repairs (Fig 3). Functional outcome data were collected by one of the authors who was not involved in the care of the patients. In the full analysis set, the results were shown in Table 1. The four groups reported significantly improved Lysholm and IKDC scores at the last follow-up relative to preoperative scores (p < 0.05). However, the macerated tears group showed least functional recovery of Lysholm and IKDC scores compared to other groups (p < 0.05). No significant difference was found between horizontal, vertical and complex tears group (p > 0.05). In the macerated tear group, Lysholm and IKDC scores at last follow-up were not significantly different between the patients with and without meniscal cysts (p > 0.05). Last follow-up mean Lysholm and IKDC scores were 84.4 ± 13.9 points (range, 78–90 points), 80.3 ± 12.1 points (range, 74–90 points), respectively, for patients without meniscal cysts; and 83.1 ± 14.2 points (range, 76–88 points), 79.2 ± 13.4 points (range, 72–86 points), respectively, for patients with meniscal cysts. In addition, there were no differences in postoperative range of motion, return to work, or return to sport/other baseline activities between the four groups (Table 1).

## Discussion

This study demonstrated that the macerated tear is common in the tear pattern of AHLM, which is also closely associated with the cyst predominantly found in lateral meniscus anterior horn. The treatment of suture repairs for the macerated tear was not superior to horizontal, vertical and complex tears, with regard to outcomes assessed at more than 12 months follow-up. Although the four groups had significant improvement in all primary outcomes, the patients with the macerated tear had no greater improvement than horizontal, vertical and complex tears.

Meniscus are made up of collagen fiber (most are type I fibers), proteoglycan, glycoprotein and elastin, and it bases on circumferential fiber and weave with radial, vertical and oblique fibers to be network structure [8,9]. In the meniscus body and lateral articular capsule (this part bearing peak load), radial fibers surround the circumferential fibers in wave shape to function buckle, which prevents meniscus from separating from the load-bearing circumferential fibers in bearing shaft (longitudinal tears) [10]. However, the AHLM insertion overlaps with the tibial anterior cruciate ligament (ACL) insertion in both the coronal and sagittal planes [11,12]. Based on the above theory, the reason that the AHLM becomes macerated is because they are impinged between the lateral femoral condyle and the anterior edge of the lateral tibial eminence. This tear morphology is clear under arthroscope, and the separation can be probed. We name the special meniscal tear morphology as macerated tear, which refers to the loose separation between circumferential fiber bundles, and this meniscal tear is defined as three and more circumferential fiber bundles can be separated by arthroscopic probe at meniscus surface or inside the injury, so as to distinguish it from the vertical, horizontal and complex tears.

Macerated tear is closely associated with meniscus cyst which is mostly found between the AHLM and the anterior cruciate ligament [13]. Differing from the even network structure of meniscus body and posterior horn, the circumferential fibers of AHLM gather to be bundles, and the loose separation between circumferential fiber bundles generate micro gap. When synovial fluid enter the gap due to one-way valve mechanism, cyst forms. Because the loose fiber bundles would cause gaps between many bundles, synovial fluid can herniate in the gaps in many directions, and thus the cysts distribute in multiple compartments and gather to be groups at varied sizes. It consists of many cysts at different sizes. This feature is anatomically explained by the macerated tear.

Optimal treatment of the macerated tears in the AHLM remains unclear. Although the macerated tears are found more often in younger patients, its tear morphology shows that it is similar to degenerative tear. After the loose separation of circumferential fiber bundles, most radial, vertical and oblique fibers which connect the bundles have damaged. Recent clinical studies have attempted to compare clinical outcomes between nonoperative management, meniscectomy, and meniscal tear repair [6,14,15]. A randomized trial showed that arthroscopic partial meniscectomy for patients without knee osteoarthritis but with symptoms of degenerative tear offers no more benefit than washout alone [16,17]. Another randomized trial showed that arthroscopic partial meniscectomy combined with physical therapy provides no better relief of symptoms than physical therapy alone in patients with degenerative tear [18,19]. Kise et al [20] reported that exercise therapy is superior to arthroscopic partial meniscectomy for knee function in middle aged patients with degenerative meniscal tears. Some systematic review and meta-analysis showed that it does not support the practice of arthroscopic surgery for middle aged or older patients with knee pain with or without signs of osteoarthritis [21–23]. In theory, loss of partial meniscus increases the risk of subsequent development of degenerative changes in the knee, and meniscal tears in the younger patients are amenable to repair [24,25]. However, few studies to date have reported on meniscal repairs for degenerative meniscal tears [5,26]. In our study, the macerated tears with meniscal repairs showed least functional recovery of Lysholm and IKDC scores as compared with that of single vertical tear, horizontal tear and complex tear. Therefore, when mechanical integrity of meniscal repair strategies cannot be achieved, experimental approaches for replacement or regeneration for meniscal tissue are required [27–30].

The present study has several limitations. First, it was a single-site study at an academic medical center. Future prospective randomized studies with appropriate sample size need to detect the morphology and tear patterns of AHLM. Furthermore, these patients also need to evaluate and treat at nonoperative management, meniscectomy, and meniscal tear repair. Last, further studies should investigate the prevalence of the macerated tears with and without AHLM cysts.

## Conclusions

In summary, the circumferential fibers of AHLM overlap with the lateral portion of the tibial ACL, and gather to be bundles. The reason that the AHLM becomes macerated is because they are impinged between the lateral femoral condyle and the anterior edge of the lateral tibial eminence. The macerated tear is the most common tear pattern of AHLM, and is also closely associated with meniscus cyst. However, feasibility of the treatment of this type of the macerated tears, especially the meniscus suture still requires further study.

## Supporting Information

S1 Video The video show that the AHLM are tears, with loose fibers in injured region and circumferential fiber bundles were separated, which can be separated by arthroscopic probe at meniscus surface or inside the injury. (MPG)
